# The B Vitamins Nicotinamide (B_3_) and Riboflavin (B_2_) Stimulate Metamorphosis in Larvae of the Deposit-Feeding Polychaete *Capitella teleta*: Implications for a Sensory Ligand-Gated Ion Channel

**DOI:** 10.1371/journal.pone.0109535

**Published:** 2014-11-12

**Authors:** Robert T. Burns, Jan A. Pechenik, William J. Biggers, Gia Scavo, Christopher Lehman

**Affiliations:** 1 Department of Biology, Tufts University, Medford, Massachusetts, United States of America; 2 Department of Biology, Wilkes University, Wilkes-Barre, Pennsylvania, United States of America; University of New South Wales, Australia

## Abstract

Marine sediments can contain B vitamins, presumably incorporated from settled, decaying phytoplankton and microorganisms associated with decomposition. Because B vitamins may be advantageous for the energetically intensive processes of metamorphosis, post-metamorphic growth, and reproduction, we tested several B vitamins to determine if they would stimulate larvae of the deposit-feeding polychaete *Capitella teleta* to settle and metamorphose. Nicotinamide and riboflavin individually stimulated larvae of *C. teleta* to settle and metamorphose, generally within 1–2 hours at nicotinamide concentrations as low as 3 µM and riboflavin concentrations as low as 50 µM. More than 80% of the larvae metamorphosed within 30 minutes at a nicotinamide concentration of 7 µM. The pyridine channel agonist pyrazinecarboxamide also stimulated metamorphosis at very low concentrations. In contrast, neither lumichrome, thiamine HCl, pyridoxine HCl, nor vitamin B_12_ stimulated larvae of *C. teleta* to metamorphose at concentrations as high as 500 µM. Larvae also did not metamorphose in response to either nicotinamide or pyrazinecarboxamide in calcium-free seawater or with the addition of 4-acetylpyridine, a competitive inhibitor of the pyridine receptor. Together, these results suggest that larvae of *C. teleta* are responding to nicotinamide and riboflavin via a chemosensory pyridine receptor similar to that previously reported to be present on crayfish chela and involved with food recognition. Our data are the first to implicate B vitamins as possible natural chemical settlement cues for marine invertebrate larvae.

## Introduction

The larvae of many benthic marine invertebrates are planktonic and are thus ‘forced to wander’ the sea until they become competent to metamorphose and locate a site suitable for settlement [1]. Particular chemical cues then promote larval settlement and subsequent metamorphosis by competent larvae of many species [2]–[4]. These chemical cues may indicate the presence of appropriate food, conspecific adults to mate with, or other environmental factors that signal the suitability of a site to live in for juveniles and adults. For example, larvae of the sea urchin *Holopneustes purpurascens* settle and metamorphose in response to histamine, which leaches away from *Delisea pulchra*, their algal food source [5]–[7]. Similarly, larvae of the tube-building polychaete worm *Phragmatopoma californica* settle and metamorphose in response to several different fatty acids that are produced by conspecific tube-dwelling adults, signaling the presence of potential mates [8]. Negative recruitment cues that prevent larvae from metamorphosing in specific areas have also been identified and can also be important in determining species distributions [9]. However, for the vast majority of marine invertebrates, the specific chemical settlement cues remain undefined.

*Capitella teleta,* previously known as *Capitella* sp. I, is a small (∼20 mm long×1 mm wide), opportunistic, deposit-feeding marine polychaete found in salt marsh sediments and in disturbed and polluted areas such as busy harbors, sewage outflows, and some regions affected by oil spills [10], [11]. Its planktonic, non-feeding, metatrochophore larvae will typically metamorphose in the presence of salt-marsh sediments, making it a convenient model for studies of substrate selection [12]–[14]. Larvae of *C. teleta* metamorphose rapidly when in the presence of salt-marsh sediment; in one study 90% of the tested larvae metamorphosed within 30 minutes of treatment [13]. The active component of the natural settlement cue contained within the salt-marsh sediment is currently unknown; however, larvae of *C. teleta* have been shown to settle and metamorphose preferentially in response to sediments with high organic content, and those with a low carbohydrate to protein ratio [15], [16]. Sediments forced through a 0.45 µm filter can still stimulate larvae to metamorphose; however, filtering the sediment to 0.22 µm removed the cue, suggesting that the cue is bound to small particulates [15]. Also, combusting the sediment at 500°C for 6 h removed the cue from the sediment: the resulting ash did not stimulate metamorphosis, supporting the notion that the cue is organic [15].

Juvenile hormones and juvenile hormone-active chemicals are known to induce settlement and metamorphosis of *C. teleta* larvae [17], [18]. Extracts prepared from marine sediments displayed juvenile hormone-activity in insect bioassays, suggesting that the chemicals in the sediments that induced settlement and metamorphosis may be similar in structure to juvenile hormones (JHs) or have juvenile hormone-activity. In addition, the induction of settlement and metamorphosis by JH was found to involve activation of protein kinase C and further activation of calcium channels [18]. Exposing *C. teleta* larvae to 400 nM calcium ionophore A23187, a membrane soluble chemical that shuttles calcium ions into cells, induced all of the larvae to metamorphose in less than one hour. Whether or not the larvae could metamorphose in calcium-free seawater however was not explored.

As has been found for some other invertebrate larvae, larvae of *C. teleta* settle and metamorphose in response to serotonin and the serotonin-reuptake inhibitor fluoxetine [19], [20], indicating that stimulation of the larval nervous system is involved in mediating the chemosensory response to chemical cues. Furthermore, different inhibitors of nitric oxide synthase such as S-methylisothiourea and aminoguanidine hemisulfate were also found to induce larval settlement and metamorphosis [20], adding *C. teleta* to a growing list of marine invertebrates, including some gastropods [21]–[23], echinoderms [24], and ascidians [25], [26] whose metamorphosis is inhibited by the presence of endogenous nitric oxide. While we currently know much about the intermediate steps of the signal transduction cascade leading to metamorphosis of *C. teleta*, we do not know which chemicals are acting as natural settlement cues for larvae of *C. teleta* or how these chemicals actually initiate this signal transduction cascade.

Larval metamorphosis, juvenile growth, and adult reproduction are all energy-requiring processes; not surprisingly the growth and reproduction of *Capitella* are affected by diet [27]. Plant nutrients have also been shown to be quickly assimilated into the growing oocytes of *Capitella* [28] and to supply adequate nutrition for larval development and subsequent metamorphosis. Dietary B vitamins are essential co-factors for biochemical energy production, and are known to be essential for the growth and development of some annelids [29]. In this respect it should be beneficial for larvae of *C. teleta* to settle and metamorphose in locations with adequate levels of these vitamins to support post-metamorphic growth. Because B vitamins are likely to be found within the marine sediments that the deposit-feeding adults of *C. teleta* consume and are required for growth and survival, we suspected that the larvae would respond to at least some B vitamins as a natural settlement cue. To test our hypothesis, we added several B vitamins individually to seawater and monitored the metamorphosis of newly-released larvae of *C. teleta*. In addition we conducted a series of experiments using various pharmacological agents to determine how these vitamins were stimulating metamorphosis.

## Results

### Effects of different B vitamins

**Figure 1:**
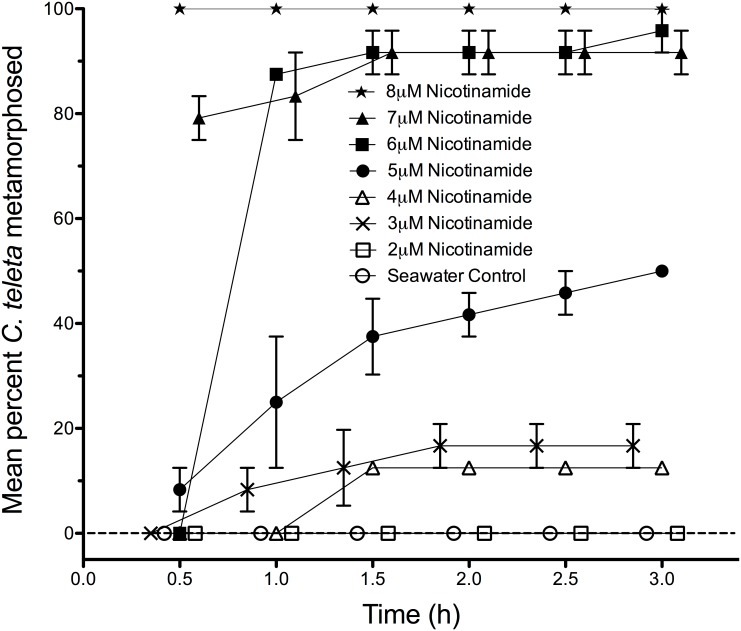
Promotion of metamorphosis by nicotinamide (vitamin B_3_) in larvae of *Capitella teleta*. Each treatment consisted of 3 replicates with 8 larvae per replicate. Larvae were placed in 30 ppt artificial seawater (Instant Ocean) containing the indicated final concentration of nicotinamide. Artificial seawater acted as a negative control. Error bars represent +/−1 s.e.m.

**Figure 2:**
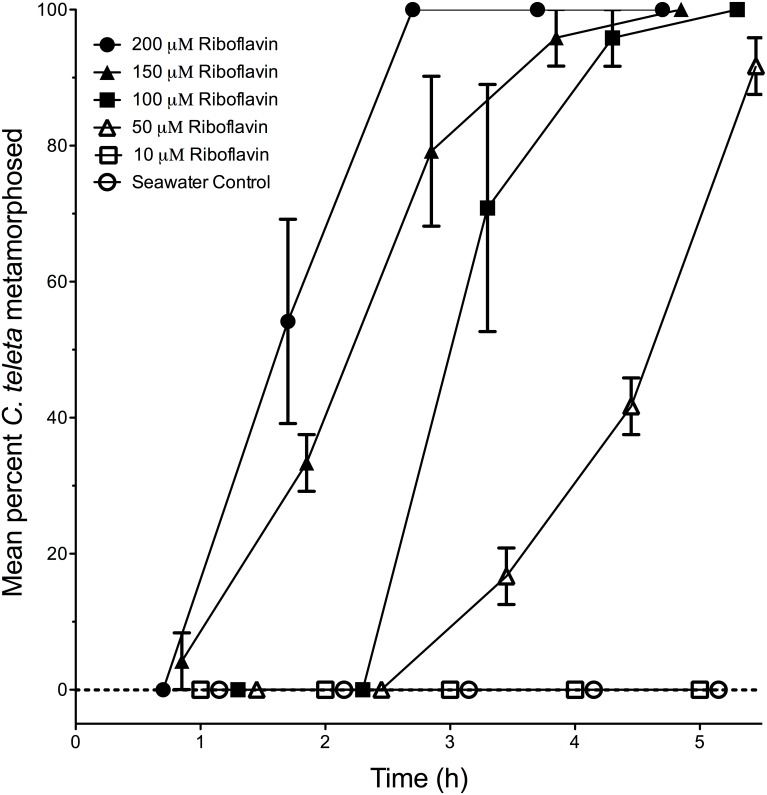
Promotion of metamorphosis by riboflavin (vitamin B_2_) in larvae of *Capitella teleta*. Each treatment consisted of 3 replicates with 8 larvae per replicate. Larvae were placed in 30 ppt artificial seawater (Instant Ocean) containing the indicated final concentration of riboflavin. Artificial seawater acted as a negative control. Error bars represent +/−1 s.e.m.

Of the 7 B vitamins tested, only riboflavin (B_2_), nicotinamide (B_3_), and nicotinic acid (another form of B_3_) stimulated metamorphosis in *C. teleta*. Thiamine HCl (B_1_), pyridoxine HCl (B_6_), biotin (B_7_), and cobalamin (B_12_) did not stimulate any metamorphosis within 24 h at the tested concentration of 500 µM (data not shown). In contrast, nicotinamide stimulated metamorphosis in a nearly dose-dependent manner at concentrations as low as 3–8 µM, with larvae initiating metamorphosis within 30 min (Figure 1). Nicotinic acid stimulated 70.8% +/−8.3 (s.e.m.) of the treated larvae to metamorphose within 24 h. Also, riboflavin concentrations of 50–200 µM stimulated nearly all larvae of *C. teleta* to metamorphose within 5 hours (Figure 2). However, the larvae did not respond to the riboflavin breakdown product lumichrome even when tested for 24 h at the maximum concentration of 1 mM (data not shown).

### Effects of pyridine channel activators and inhibitors

**Figure 3:**
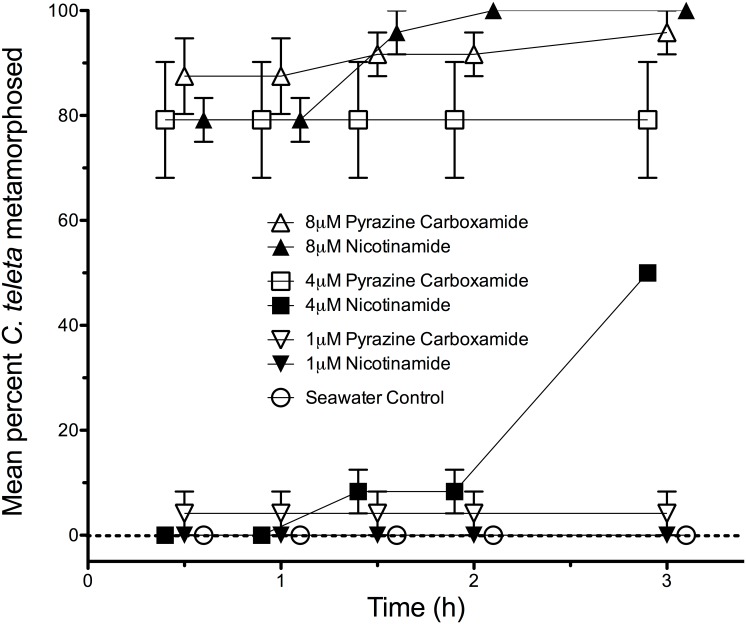
The effects of pyrazinecarboxamide and nicotinamide on metamorphosis of *C. teleta* when tested equal concentrations. Each treatment consisted of 3 replicates with 8 larvae per replicate. Artificial seawater (Instant Ocean) acted as a negative control. Error bars represent +/−1 s.e.m.

Because concentrations of nicotinamide as low as 3–6 µM stimulated larvae of *C. teleta* to metamorphose quickly in our experiments, we hypothesized that larvae of *C. teleta* were sensing nicotinamide via a pyridine activated ion channel, something first characterized in the walking legs of the crayfish *Austrapotamobius torrentium* [30]–[32]. We therefore compared dose responses between nicotinamide and pyrazinecarboxamide, an agonist of the pyridine-activated ion channel, at 1 µM, 4 µM, and 8 µM. Another commonly occurring nutritive chemical, beta nicotinamide adenine dinucleotide (β-NAD), also stimulated the nicotinamide-activated ion-channel to open, with half-maximal rate of opening (K_M_) at 1 mM [31]. We therefore treated larvae of *C. teleta* with β-NAD at the concentrations of 0.5, 1, and 5 mM to determine if β-NAD also stimulated metamorphosis. As shown in Figure 3, the pyridine-activated ion channel agonist pyrazinecarboxamide was found to also stimulate larvae of *C. teleta* to metamorphose, and did so at similar concentrations as nicotinamide. Although both chemicals stimulated larvae of *C. teleta* to metamorphose in a dose-dependent fashion, pyrazinecarboxamide stimulated more individuals (about 70%) to metamorphose at 4 µM than did nicotinamide at the same concentration (Figure 3). β-NAD, however, did not stimulate any metamorphosis at 0.5, 1, or 5 mM.

### Effects of calcium free seawater

**Figure 4:**
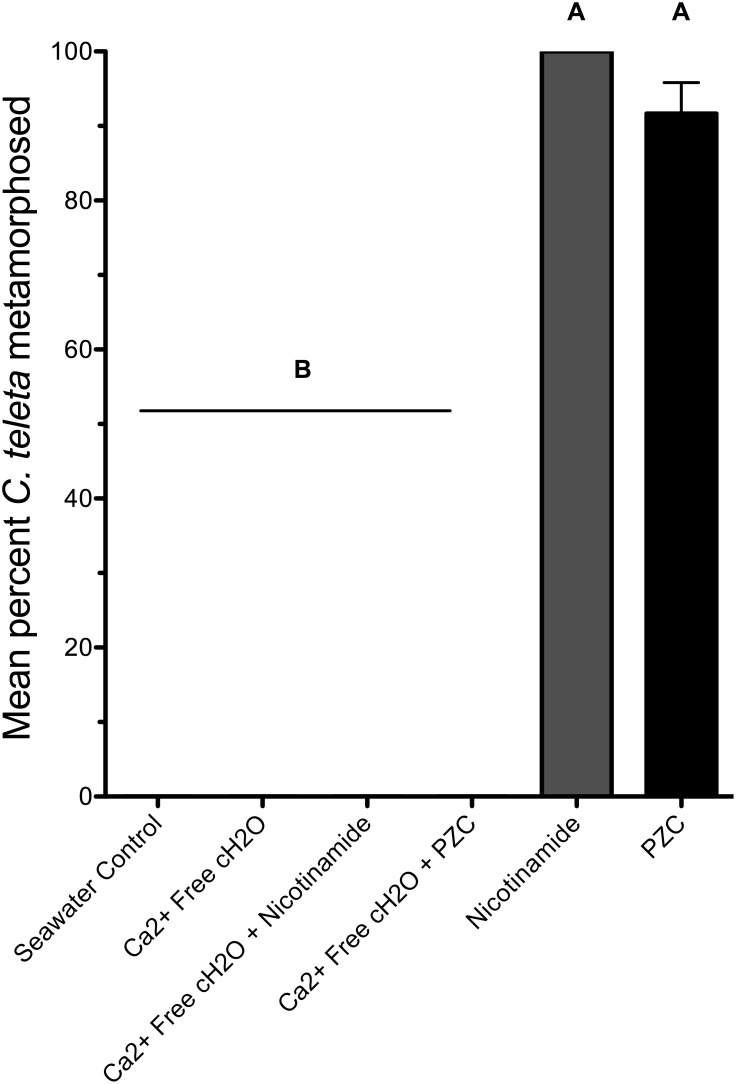
The effect of calcium-free seawater on metamorphosis of *C. teleta* treated with nicotinamide or pyrazinecarboxamide. Larvae were acclimated to calcium-free seawater for one hour before being transferred to a solution of calcium-free seawater and either nicotinamide or pyrazinecarboxamide (PZC) at 40 µM for 24 h. Each treatment consisted of 3 replicates with 8 larvae per replicate. Artificial seawater (Instant Ocean) acted as a negative control. Letters indicate significant (P<0.05) differences as determined by a Bonferroni post-hoc test. Error bars represent +1 s.e.m.

To determine if an influx of extracellular calcium is required for metamorphosis to proceed when pyridine-activated ion channels open, larvae of *C. teleta* were treated with nicotinamide or pyrazinecarboxamide while bathed in either normal ASW(control) or calcium-free ASW. Pre-treating the larvae in calcium-free ASW inhibited the metamorphic response of *C. teleta* larvae to both nicotinamide and pyrazinecarboxamide even at the high concentration of 40 µM (one-way ANOVA with Bonferroni post-hoc comparisons, F(5,12) = 234.4, p<0.001) (Figure 4). Larvae in calcium-free ASW settled to the bottom of the dish within a few minutes after the addition of nicotinamide or pyrazinecarboxamide; they then remained stationary, but did not metamorphose over the 24 h observation period. Control larvae in calcium-free ASW continued to swim normally and neither settled nor metamorphosed. At least 90% of larvae exposed to nicotinamide or pyrazinecarboxamide in ASW metamorphosed, indicating that most larvae were competent to metamorphose at the start of this experiment.

### Effects of 4-acetylpyridine

**Figure 5:**
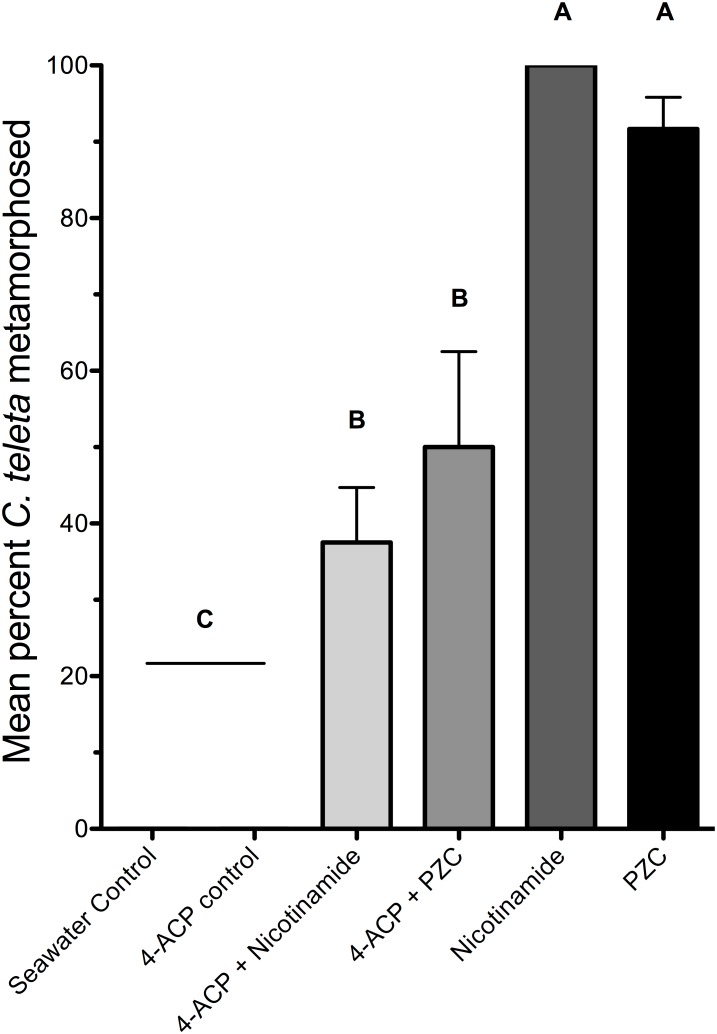
The effect of 4-acetylpyridine on metamorphosis of *C. teleta* in the presence of nicotinamide or pyrazinecarboxamide. Larvae were acclimated to 200 µM 4-acetylpyridine (4-ACP) for one hour before being transferred to a solution of 200 µM 4-acetylpyridine and either nicotinamide or pyrazinecarboxamide (PZC) at 40 µM for 24 h. Each treatment consisted of 3 replicates with 8 larvae per replicate. Artificial seawater (Instant Ocean) acted as a negative control. Letters indicate significant (P<0.05) differences as determined by a Bonferroni post-hoc test. Error bars represent + s.e.m.

**Figure 6:**
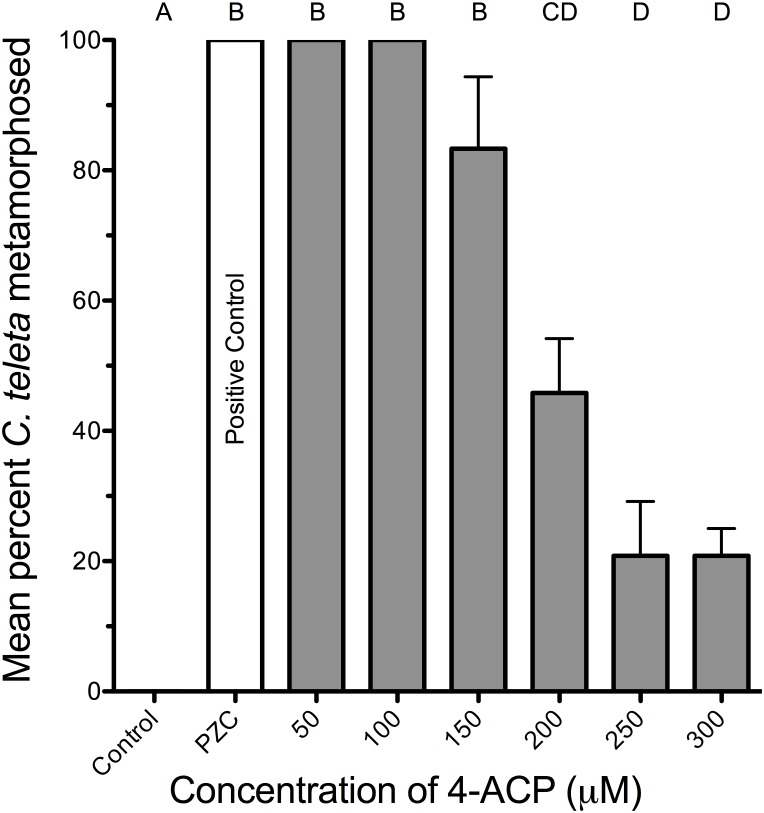
The inhibitory dose response of 4-acetylpyridine on metamorphosis of *C. teleta* after 24 h. Larvae were acclimated to the listed concentrations of 4-acetylpyridine (4-ACP) for one hour before being transferred to a solution of the same concentration of 4-acetylpyridine and pyrazinecarboxamide at 40 µM. Each treatment consisted of 3 replicates with 8 larvae per replicate. Artificial seawater (Instant Ocean) acted as a negative control. The PZC treatment acted as a positive control containing only 40 µM pyrazinecarboxamide and artificial seawater. Letters indicate significant (P<0.05) differences as determined by a Bonferroni post-hoc test. Error bars represent +1 s.e.m.

4-acetylpyridine, a specific competitive antagonist of the pyridine-activated ion channel, was also tested on larvae of *C. teleta* in the presence of either nicotinamide or pyrazinecarboxamide [32]. Treating larvae with 200 µM 4-acetylpyridine inhibited them from metamorphosing in the presence of either 40 µM nicotinamide or 40 µM pyrazinecarboxamide (one-way ANOVA with Bonferroni post-hoc comparisons, F(5,12) = 69.52, p<0.001) (Figure 5). Only about half as many larvae metamorphosed within 24 h when exposed to both 4-acetylpyridine and either nicotinamide or pyrazinecarboxamide, indicating that the inhibitory concentration allowing for 50% settlement and metamorphosis (IC_50_) is about 200 µM. No larvae exposed to both 4-acetylpyridine and either nicotinamide or pyrazinecarboxamide metamorphosed within the first 5 h of the experiment (data not shown) and no control larvae exposed to 200 µM 4-acetylpyridine alone settled or metamorphosed within 24 h. Again, the high response of larvae in the positive controls shows that most of the larvae were metamorphically competent when tested. Also, larvae were inhibited from metamorphosing in response to pyrazinecarboxamide in a dose-dependent fashion when tested with increasing doses of 4-acetylpyridine (one-way ANOVA with Bonferroni post-hoc comparisons, F(7,16) = 53.92, p<0.001) (Figure 6).

### Effects of ketanserin

**Figure 7:**
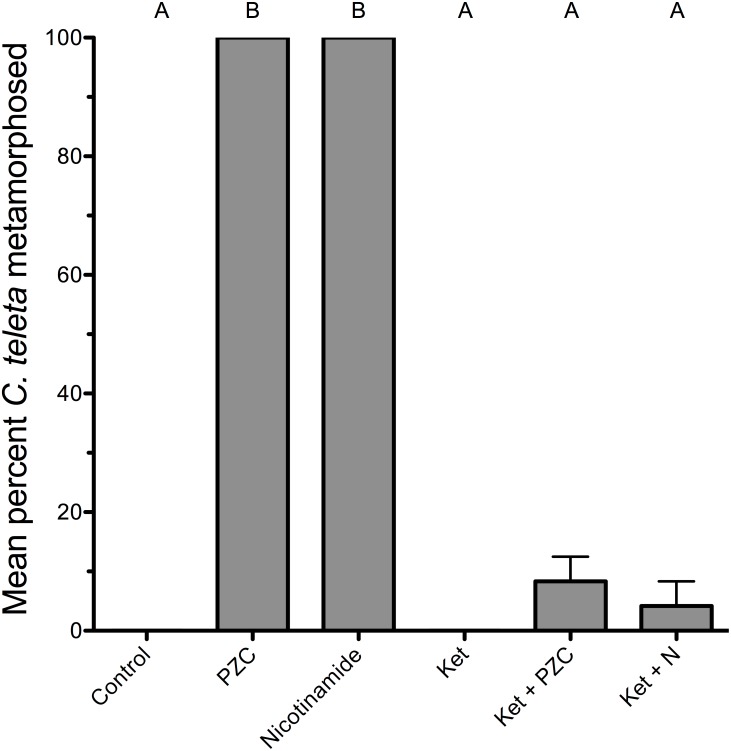
The effect of ketanserin on metamorphosis of *C. teleta* in the presence of nicotinamide or pyrazinecarboxamide. Larvae were acclimated to 2 µM ketanserin for three hours before being transferred to a solution of 2 µM ketanserin and either nicotinamide or pyrazinecarboxamide at 40 µM for 24 h. Each treatment consisted of 3 replicates with 8 larvae per replicate. Artificial seawater (Instant Ocean) acted as a negative control. Letters indicate significant (P<0.05) differences as determined by a Bonferroni post-hoc test. Error bars represent + s.e.m.

Sensory information relayed by sensory chemoreceptors such as olfactory receptor neurons usually involves sensory transduction through synapses within the central nervous system. In order to assess the involvement of the serotonergic nervous system in mediating the response of the larvae to nicotinamide and pyrazinecarboxamide, the larvae were first pre-exposed to ketanserin, an inhibitor of serotonin 5-HT_2B_ receptors, before induction by nicotinamide and pyrazinecarboxamide. Previous results have demonstrated that ketanserin blocks the response of *Capitella* larvae to natural mud sediment chemical cues, and also to the nitric oxide synthase inhibitors N-methyl-L-arginine and S-methylisothiourea [19], [20], indicating the involvement of the serotonergic nervous system in mediating the settlement and metamorphosis of *C. teleta*. Pre-exposing *C. teleta* larvae to ketanserin inhibited most of the larvae of *C. teleta* from metamorphosing in response to both nicotinamide and pyrazinecarboxamide (one-way ANOVA with Bonferroni post-hoc comparisons, F(5,12) = 122.5, p<0.001) (Figure 7). No control larvae exposed to 2 µM ketanserin alone settled or metamorphosed within 24 h; however, all of the larvae exposed to the positive controls of nicotinamide and pyrazinecarboxamide alone metamorphosed in less than an hour, indicating that most larvae were competent when tested.

## Discussion

Overall, these data support our hypothesis that at least some B vitamins may act as chemical cues for habitat selection by the larvae of *C. teleta* and may stimulate the larvae of this species to metamorphose in the field. In particular, nicotinamide (B_3_), nicotinic acid (B_3_), and riboflavin (B_2_) stimulated larvae of *C. teleta* to metamorphose, whereas thiamine HCl, pyridoxine HCl, biotin, vitamin B_12_, and the riboflavin breakdown product lumichrome did not. It may be advantageous for marine larvae to settle and metamorphose in response to environmental riboflavin and nicotinamide, as riboflavin is required for the synthesis of FAD, and nicotinamide serves as a building block for the synthesis of NAD; both products are important electron carriers for the electron transport chain, ATP production, and other aspects of cellular metabolism. Substantial concentrations of these vitamins may serve as an adaptive signal that the local environment is suitable for the energetically intensive processes of juvenile development and adult reproduction. In this regard, B vitamins present in ocean waters have been demonstrated to be important for the growth of phytoplankton, zooplankton, and larger marine invertebrates [33]–[35]. However, even if B vitamins are contained within marine sediments, they may not always be bioavailable to deposit feeders.

Although the stimulation of settlement and metamorphosis by vitamins seems not to have been investigated in other marine invertebrate species, the larvae of several other marine invertebrates have previously been demonstrated to settle and metamorphose in response to certain vitamin derivatives [2]. For example, larvae of the ascidian *Halocynthia roretzi* will settle and metamorphose in response to lumichrome, a photocatalytic breakdown product of riboflavin, although they did not respond to riboflavin itself [36]. This inducer was found to be naturally biosynthesized by the larvae and adults of *Halocynthia*. Lumichrome, however, did not induce settlement and metamorphosis in other ascidian species, and also did not stimulate metamorphosis in our study, although the larvae of *C. teleta* did respond to riboflavin. In other studies, larvae of the hydroid *Coryne uchidae* were found to settle and metamorphose in response to delta-tocopherol epoxides, compounds related in structure to the alpha-tocopherol (vitamin E) produced by the brown algae *Sargassum tortile* [37].

The vitamin requirements for the growth, reproduction, and development of *C. teleta* have so far been unexplored, to our knowledge; however, as previously mentioned, we know that growth, reproduction, and development of *C. teleta* depend strongly on diet and organic content of sediments [27], [38], and that nutrients such as algal pigments, including carotenoids, are rapidly assimilated into developing oocytes [28]. As previously mentioned, larval settlement of *C. teleta* depends on the organic content of sediments, with larvae preferring sediments with a low carbohydrate to protein ratio [15], [16]. Very little is known about marine annelid nutrition and vitamin requirements; however, an absolute requirement for riboflavin and nicotinamide, along with 6 other vitamins, was demonstrated using artificial culture media for the oligochaete annelid *Enchytraeus fragmentosus* [29].

We do not currently know the concentrations of riboflavin and nicotinamide present naturally in the environments that larvae of *C. teleta* settle and metamorphose in. It is likely, however, that recently deposited sediments, particularly those near sewage outflows, contain high levels of these compounds, sufficient to induce metamorphosis. Many phytoplankton species are known to contain large concentrations of B vitamins within their cells [39]. Several marine microorganisms, such as the bacteria *Shewanella spp*. [40] and *Micrococcus luteus* [41], and the yeast *Candida* [42], are also known to produce and release large amounts of riboflavin particularly when grown as biofilms. Remnants of settled, decomposing phytoplankton and the other microbes taking part in decomposition processes are likely to be a large component of marine sediments, which should therefore contain high concentrations of B vitamins. Indeed, biotin (B_7_), thiamine (B_1_), and vitamin B_12_ have been all identified in Pacific marine sediments [43]. Additional studies will be required to determine if riboflavin and/or nicotinamide are present in salt marsh sediments and sediments near sewage outfalls at the low concentrations that we have found to be inductive.

Although the biochemical and sensory mechanisms through which the larvae of *C. teleta* respond to nicotinamide and riboflavin still need more investigation, our results suggest that larvae may be sensing nicotinamide with chemosensory pyridine receptors similar to those previously found in chemosensory sensilla on the walking legs of crayfish, and which are involved in the foraging behavior of those animals [30]–[32]. The inhibitory effects of 4-acetylpyridine on chemosensation of nicotinamide support this suggestion, as do the stimulatory effects of pyrazinecarboxamide on the larvae of *C. teleta*, a powerful agonist of these pyridine-activated ion channels [32]. In crayfish, pyrazinecarboxamide was found to be the most effective inducer, followed by nicotinamide; we have found that this is also the case for larvae of *C. teleta* (Table 1) [32]. Also, the IC_50_ for 4-acetylpyridine was found to be approximately 200 µM for *C. teleta* compared with a K_I_ of 70 µM for the crayfish *A. torrentium* [32]. This difference in effective concentrations for 4-ACP may at least be partly due to the fact that Hatt et al. [32] explored the effects of 4-ACP using isolated chemosensory neurons and electrophysiological techniques, whereas the effects on *C. teleta* were explored in *in vivo* studies in which the chemical had to also penetrate the larvae. Interestingly, larvae did not metamorphose when exposed to β-NAD at concentrations as high as 5 mM. While nicotinamide, pyrazinecarboxamide, and nicotinic acid stimulated larvae to metamorphose at concentrations similar to those that stimulated action potentials in the crayfish, it may be possible that other pyridines such as β-NAD do not bind efficiently to this receptor at similar concentrations for both species. It would be interesting in this regard to also try a patch-clamp technique on the *C. teleta* larvae to demonstrate the presence of pyridine receptors.

**Table 1:** The lowest stimulatory concentrations of pyridines that induced larvae of *C. teleta* to metamorphose compared with the K_M_ of these same chemicals on the pyridine-activated ion channel of the crayfish *A. torrentium.*

	Species	
Chemical	*C. teleta*	*A. torrentium*
Pyrazinecarboxamide	1 µM	1.5 µM
Nicotinamide	3 µM	10 µM
Nicotinic acid	1 mM	>1 mM
4-acetylpyridine	200 µM	70 µM

The concentration of 4-acetylpyridine that inhibited 50% of the larvae of *C. teleta* is also compared with the IC_50_ of 4-acetylpyridine for the crayfish (adapted from Hatt and Schmiedel-Jacob, 1984).

Each year, more ligand-gated ion channels have been characterized as having roles in animal’s taste reception [44], although the presence of pyridine receptors used for chemodetection in other animals besides crustaceans to our knowledge has not been reported. Signal transduction carried out by these ion channels may be calcium dependent [45]. Our results indicating that calcium-free seawater inhibited metamorphosis in response to nicotinamide also therefore agree with these data. Although larvae of *C. teleta* did not metamorphose in the presence of nicotinamide or pyrazinecarboxamide in calcium-free seawater in our study, they did settle to the bottom in those solutions within several minutes. Most likely, the nicotinamide or pyrazinecarboxamide ligand is binding to the channel and opening it; however, without calcium ions entering through the channel, the signal transduction pathway leading to metamorphosis is probably stalled shortly after initiation. These results parallel the results of Biggers and Laufer [18], showing that settlement and metamorphosis of *C. teleta* can be induced by very low concentrations of the calcium ionophore A23187.

Our results using the 5-HT_2B_ receptor inhibitor ketanserin suggest that the response to nicotinamide and riboflavin requires participation of the serotonergic nervous system as well, since ketanserin inhibited this response. These data suggest that the larvae detect nicotinamide and riboflavin in the environment via chemosensory neurons likely present in the chemosensory cilia noted by Eckelbarger and Grassle [46]. Sensory depolarization of these receptors may lead to the release of serotonin either directly by the chemosensory neurons or indirectly through synaptic activation of other serotonergic neurons. Nitric oxide may play a role as a secondary messenger between the influx of ions caused by the opening of nicotinamide activated ion channels and serotonergic signaling. Larvae of *C. teleta* did not metamorphose in the presence NOS inhibitors when treated with ketanserin, a serotonin 5-HT_2B_ receptor antagonist [20]. Serotonin binding to receptors then most likely leads to settlement and metamorphosis. In this regard, the serotonin 5-HT_2B_ receptor is known to be a metabotropic receptor involving G-protein activation, secondary messengers, calcium channel activation, and downstream kinase activation such as through mitogen activated protein kinases (MAPK) involved in mediating gene expression [47]. Other chemosensory structures as reviewed by Lindsay [48] may be also be involved in the detection of nicotinamide and riboflavin by these larvae.

Larvae of *C. teleta* may be foraging for an environment containing nutrient-rich sediment prior to metamorphosing and thereby may be exhibiting foraging behavior in choosing habitats for settlement and metamorphosis. By responding to the B vitamins nicotinamide and riboflavin as chemical settlement cues, they could be ensuring that a selected environment contains the proper vitamins to successfully grow and reproduce. As deposit feeders, *C. teleta* can utilize these vitamins only if they are contained within marine sediments. Here, larvae of *C. teleta* seem to be mirroring how adult crustaceans such as crayfish and lobsters sense nutrients within sediments via chemosensory hairs attached to their legs or antennules. The crayfish *A. torrentium* can sense nicotinamide and other pyridines while the spiny lobster *Panulirus argus* can sense ATP using their antennules [49]. Interestingly, the crayfish *Astacus astacus* has been demonstrated to have specific internal oesophageal receptors for nicotinamide [50], and the external receptors on the walking legs and antennules of crustaceans may represent primitive fore-runners of internal chemoreceptors such as the oesophageal receptors [49]. To our knowledge, the present paper is the first demonstration of the presence of nicotinamide or riboflavin receptors in an annelid or in invertebrate larvae.

## Materials and Methods

### Ethics statement

No permissions were required to collect sediment from the Little Sippewissett salt marsh (West Falmouth, MA) (41°34′34.4″N 70°38′08.6″W). This activity did not involve endangered or protected species.

### Care and maintenance of larvae

Adults of *Capitella teleta* were provided by Dr. Judith Grassle (Rutgers University) and maintained in the lab since 2012 in 9 cm glass dishes containing 30 psu Instant Ocean artificial seawater (hereafter called ASW) at 18°C. Sediment collected from the Little Sippewissett salt marsh (West Falmouth, MA) was sieved through a 1 mm wire mesh screen, frozen for at least 24 h, aerated, and provided as food *ad libitum* [13], [14]. Cultures were searched for brooding females every 2–3 days; brooding individuals were then transferred individually to 6 cm glass dishes containing ASW. Dishes containing brooding females were checked daily for swimming larvae; larvae were then pipetted into a separate, clean 6 cm glass dish containing ASW. All experiments used larvae released only within the previous 24 hours. *C. teleta* larvae are usually competent to settle and metamorphose within minutes after their escape from the brood tube [11], [14], so that all larvae should have been competent at the start of our experiments.

### General bioassay protocol

All experiments were conducted using either 12-well tissue-culture plates or 3 cm glass dishes, with each well or bowl containing 4 mL of a vitamin solution in ASW, or a negative control (ASW). Each experiment included 3 replicates per treatment, with 8 larvae per replicate. To avoid diluting the final treatment solution, larvae were first pipetted into an intermediate bath of treatment solution (∼20 mL) before being pipetted into the final treatment vessel. Dishes were examined every 30 or 60 minutes under 20–50x magnification to count the number of newly metamorphosed juveniles. Larvae of *C. teleta* were considered to have metamorphosed when they elongated, lost their prototroch and telotroch cilia, and began crawling on the bottom of the vessel. All experiments were conducted at room temperature (20–23°C) under laboratory fluorescent lighting.

### Testing the effects of B vitamins and other chemicals on larval metamorphosis

Thiamine HCl (B_1_), riboflavin (B_2_), nicotinamide (B_3_), nicotinic acid (another form of B_3_), pyridoxine HCl (B_6_), biotin (B_7_) and cobalamin (B_12_) were obtained from Sigma Chemical Co. Thiamine HCl, nicotinic acid, pyridoxine HCl, biotin, and cobalamin were all tested on larvae of *C. teleta* at 500 µM. Nicotinic acid was also tested at 1 mM. Riboflavin was tested on larvae of *C. teleta* at 10, 50, 100, 150, and 200 µM. Nicotinamide was tested on larvae of *C. teleta* at 2, 3, 4, 5, 6, 7, and 8 µM. The concentrations tested for nicotinamide and riboflavin were based on preliminary pilot studies. The photo-degradation product of riboflavin, lumichrome, was obtained from Sigma Chemical and tested at concentrations of 100, 500, and 1000 µM. The pyridine receptor antagonists and agonists 4-acetylpyridine, pyrazinecarboxamide, and β-NAD were all purchased from Sigma Chemical Co. and tested in final concentrations as stated in results. The serotonin receptor antagonist ketanserin was purchased from Sigma Chemical Co. and prepared as a 10 mM stock solution in ethanol.

### Testing the effects of 4-acetylpyridine, calcium-free ASW, and ketanserin on metamorphosis

Larvae were acclimated to ASW containing 200 µM 4-acetylpyridine for one hour and then transferred to a treatment containing either 200 µM 4-acetylpyridine + 40 µM nicotinamide, 200 µM 4-acetylpyridine + 40 µM pyrazinecarboxamide, or 200 µM 4-acetylpyridine as a negative control. Larvae that were not acclimated to 4-acetylpyradine were transferred to positive controls containing either 40 µM nicotinamide or 40 µM pyrazinecarboxamide.

Calcium-free ASW was prepared by omitting calcium chloride from the recipe given in [51]. Larvae that were in calcium-free ASW treatments were acclimated to calcium-free ASW for one hour and then were transferred to treatments containing either calcium-free ASW+40 µM nicotinamide, calcium-free ASW + 40 µM pyrazinecarboxamide, or calcium-free ASW only (negative control). Larvae that were not acclimated to calcium-free ASW were transferred to positive controls containing either 40 µM nicotinamide or 40 µM pyrazinecarboxamide to assess metamorphic competence.

Larvae were acclimated to ASW containing 2 µM ketanserin for three hours and then transferred to a treatment containing either 2 µM ketanserin + 40 µM nicotinamide, 2 µM ketanserin + 40 µM pyrazinecarboxamide, or 2 µM ketanserin as a negative control. Larvae that were not acclimated to ketanserin were transferred to positive controls containing either 40 µM nicotinamide or 40 µM pyrazinecarboxamide.

### Data analysis

In order to determine if calcium-free seawater, 4-acetylpyridine, or ketanserin influenced the number of *C. teleta* larvae that metamorphosed in the presence of nicotinamide or pyrazinecarboxamide, a one-way analysis of variance was conducted on the numbers of *C. teleta* that had metamorphosed by 24 h after treatment. If the results of an ANOVA were significant, Bonferroni’s post-hoc test was conducted to determine which treatments were significantly different from each other. For each ANOVA, each replicate was the arcsine transformed ratio of individuals that had metamorphosed at that timepoint. It is important to note that, although the figures represented in this paper reflect the percent of *C. teleta* larvae that had metamorphosed, all statistical analyses were conducted with arcsine transformed ratio of the larvae that had metamorphosed. All statistical analyses were carried out with GraphPad Prism version 5.0.
